# Tumor Recurrence and Follow-Up Intervals in Oral Squamous Cell Carcinoma

**DOI:** 10.3390/jcm11237061

**Published:** 2022-11-29

**Authors:** Sebastian Blatt, Maximilian Krüger, Kawe Sagheb, Marie Barth, Peer W. Kämmerer, Bilal Al-Nawas, Keyvan Sagheb

**Affiliations:** 1 Department of Oral- and Maxillofacial Surgery—Plastic Surgery, University Medical Centre, Augustusplatz 2, 55131 Mainz, Germany; 2 Department of Prosthetic Dentistry, University Medical Centre, Augustusplatz 2, 55131 Mainz, Germany

**Keywords:** oral cancer, prognosis, squamous cell carcinoma, recurrence interval, differentiation

## Abstract

Tumor recurrence in oral squamous cell carcinoma (OSCC) is frequent. However, no consensus about follow-up interval is available. The aim of this study was to analyze the recurrence pattern, detection method and associated parameters for possible risk stratification. Histopathological and epidemiological features were obtained retrospectively and correlated with tumor recurrence and overall survival, distant and lymph node metastases. A total of 760 patients were included, of which 216 patients showed tumor recurrence (mean after 24 ± 26 months). Within the first 12 months, 24% of the recurrences were detected. The primary detection method was clinical examination (*n* = 123, 57%). Tumor recurrence significantly correlated with advanced histopathological grading (G2/3 vs. G1, *p* < 0.000) and lymph node metastasis (*p* = 0.004). Tumor recurrence was frequent. Clinical examination was the primary detection method and manifestation within the first 6–12 months was high. The degree of histopathological grading may be useful for risk stratification.

## 1. Introduction

Head and neck squamous cell carcinoma (HNSCC) is the sixth most common cancer worldwide; the sub-entity of squamous cell carcinoma of the oral cavity (OSCC) accounts for approximately 300,000 cases and 145,000 deaths per year worldwide [1]. After primary curative treatment of OSCC, recurrence can be detected in about 20% of cases; 76% of recurrences occur within the first two years [2]. Others even state recurrence rates of up to 86% within the first year [3]. Although multiple national and international guidelines and recommendations of different working groups provide proposals for OSCC follow-up regime, there is no consensus regarding timing and frequency of consultations and no established standard for examination routines [4,5,6,7]. In the German guideline, a quarterly interval of follow-up visits even in symptom-free patients in the first two years and half-yearly after 3–5 years is advised [8]. Even so, mostly self-reported symptoms and not detection during regular follow-up visits of OSCC patients led to diagnosis of recurrence in up to 78% of the cases [6]. In accordance, there is limited evidence concerning the effectiveness of follow-up after primary OSCC treatment and only low-level evidence suggests an improvement in oncologic outcomes by close follow-up. Therefore, for development of evidence-based follow-up advice after OSCC, future research should focus on risk stratification [7].

Today, the most common prognostic factors for overall survival and tumor recurrence are lymph node metastasis, tumor size and resection status [9,10], whereas a correlation between histopathological grading and overall and disease-free survival is discussed with controversial results. Especially for early tumor stages, histopathological grading is found to be of little predictive value [11].

The aim of this study was to analyze recurrence frequency and associated parameters of patients with OSCC. We hypothesize that histopathological features, such as grading, are correlated with tumor recurrence and may represent a possible marker for risk stratification. This way, visit interval could be personalized for high-risk patients.

## 2. Materials and Methods

### 2.1. Study Cohort

Within this retrospective study, clinical records of 831 consecutive patients with histopathological diagnosis of primary oral squamous cell carcinoma (OSCC) treated at the Department of Oral and Maxillofacial Surgery Mainz, Germany between 2000 and 2015 were analyzed. The exclusion criteria were malignancies other than OSCC, distant metastasis at the time of primary presentation, OSCC treated with primary radio(-chemo) therapy as well as OSCC that has received therapy before. All patients were treated in accordance to current guideline recommendations and treatment approaches were evaluated by interdisciplinary tumor boards. If general conditions did not allow primary surgery or the tumor was too extensive to be resected without significant mutilations, patients were assigned to either radiation, chemotherapy or in combination, and excluded from the study. Patients eligible for surgery received tumor resection and at least a selective neck dissection of the ipsilateral site in neck level I–III. Histologically, safe resection margins >5 mm were defined as R0. Epidemiological features (gender, age, risk profile for either alcohol, tobacco or both) and histopathological data (T (=tumor) N (=node) M (=metastasis) staging, histology resection margin and grading) were obtained from all patients. For grading, degree of keratinization, number of nuclear polymorphisms as well as mitoses, and pattern of invasion were evaluated as previously described [12].

After cancer treatment, follow-up time, frequency of visits, tumor recurrence and disease free as well as overall survival were analyzed. Regular follow-up consisted of clinical visits every 4 weeks until 6 months postoperatively, then an interval of 2–3 months was chosen until 2 year postoperatively, followed by a one year interval afterwards. Radiographical examination consisted of CT scan with contrast of the head and neck region after 3–6 months as well as 1 year after initial operation. Subsequently, CT scan with contrast was performed yearly. In cases with allergy to contrast agent, MRI of the head and neck region was done within the same intervals. In addition to clinical and radiological inspection as indicated, no adjunctive chair-side test was performed to detect possible recurrence. Recurrence was defined as follows: (1) local recurrence—recurrence at the same anatomic site within 5 years after primary treatment; (2) regional recurrence—lymph node metastases of the neck within 5 years after primary treatment; (3) distant metastases—metastases elsewhere in the body, e.g., the lungs; and (4) second cancer of the oral cavity—carcinomas elsewhere in the aerodigestive tract within 5 years after primary treatment or recurrence at the same anatomical site 5 years after primary treatment. Recurrence therapy was defined as surgical resection, radiation alone, chemotherapy alone or the combination of both or in combination with surgery. Recurrence interval was defined as the duration from the end of initial treatment to the time of recurrence confirmed by pathological examination after incisional biopsy. The study was conducted within the Helsinki Declaration of Human Rights and with approval of the local ethics committee (number 2018-13844).

### 2.2. Statistics

For statistical analysis, SPSS 23.0 for Windows (IBM Deutschland GmbH, Ehningen, Germany) was used. The results were shown as arithmetic means ± standard error of the mean (SEM) and rounded to one decimal digit. Before testing, all variables were evaluated for normal distribution applying the Shapiro–Wilk test. Pearson chi-Square test and Fisher’s exact test as well as multivariate analysis (ANOVA) were accomplished to detect possible risk factors for tumor relapse, overall and disease-free survival. Here, Kaplan–Meier plots in combination with log-rank Mantel–Cox regression were used. A *p*-value ≤ 0.005 was defined as statistically significant.

## 3. Results

Of a total of 831 patients, 71 patients did not meet the inclusion criteria due to histological evidence of carcinoma in situ (*n* = 14), distant metastasis at the time of presentation (*n* = 9) and, because of far advanced tumor stage, nonsurgical approach but primary radiochemotherapy (*n* = 48, Table 1). The median age was 62 ± 13 years. Of the cohort, 258 patients (34%) were female and 502 (66%) were male. 569 (75%) patients showed a positive risk profile for alcohol and tobacco at initial staging. Here, the most frequent was combination of smoking and alcohol abuse (49%), before smoking alone (13%) and alcohol abuse (13%) alone. Primary tumor sites were floor of the mouth (30%), tongue (27%), oral mucosa of the mandible (22%) or the maxilla, respectively (15%), and cheek (6%). Tumor size was advanced (T3/T4) in 29% of the cases, where the vast majority displayed a smaller primary tumor size (T1/T2, 71%). Histopathological grading was moderately and poorly differentiated (G2/G3) in 635 cases (83%). The resection margin >5 mm (defined as in sano resection) was achieved in 85% of the cases. A close resection margin of <5 mm was shown in 14% of cases and 1% (7 cases) were intraoperatively defined as R2-status with non-resectable tumor spread. For therapy options, surgery only was performed in 423 (56%) patients, where 146 patients (19%) needed adjuvant radiation, 191 (25%) adjuvant radiochemotherapy because of far advanced tumor stages.

A total of 216 (28%) patients showed OSCC recurrence in the median follow up time of 40 ± 40 months (Table 2). Mean interval until tumor recurrence was 24 ± 26 months. Recurrence types were local recurrence (10%), second cancer of the oral cavity (7%), lymph node metastases (6%) and distant metastases (3%). Seven patients (1%) showed a combination of local and lymph node (*n* = 3) or distant metastases (*n* = 4). The vast majority of the patients developed one recurrence (*n* = 181, 24%); 35 patients displayed more than one manifestation (5%). Within the first 12 months after primary treatment, 24% were detected, after 24 months, 50% of all recurrences became manifest. After three years, 76% of recurrences were detected. In the fifth year of follow-up, 89% of recurrences were manifested. The latest recurrence was found 135 months after diagnosis.

**Figure 1:**
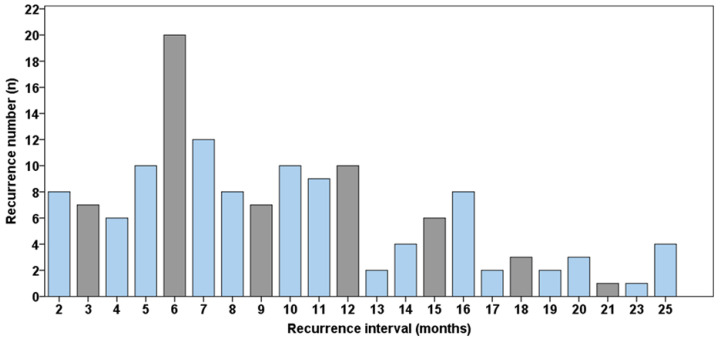
Distribution of recurrence interval of the relapse cases (*n* = 216).

For detection of tumor recurrence, clinical examination found the majority of tumor manifestations (57%, *n* = 123), followed by radiological diagnosis with CT (27%, *n* = 57), ultrasonography (8%, *n* = 18) and self-reported symptoms (8%, *n* = 18). In the clinical examination, recurrences presented inter alia as ulcers, lumps, plaque and eroded areas. Self-reported symptoms were pain, burning sensation, difficulty chewing and swallowing as well as globus sensation. Figure 1 shows the time points when recurrences were detected during follow-up.

Pearson chi-Square test and Fisher’s exact test revealed a strong statistically significant correlation of advanced histopathological grading and tumor recurrence (G2/3 vs. G1, *p* < 0.001). In addition, lymph node metastasis was likewise associated with tumor recurrence (*p* = 0.004). Tumor size had no significant influence on recurrence (*p* = 0.502) as well as positive resection status (*p* = 0.600). In accordance, the multivariate analysis testing for age and TNM status, revealed a strong association between advanced grading and recurrence (*p* = 0.001), and for positive lymph node status (*p* = 0.003) and recurrence. Furthermore, tumor size (*p* = 0.503), gender (*p* = 0.366), risk factors (*p* = 0.312) and a positive resection status (*p* = 0.380) had no influence on recurrence.

**Figure 2:**
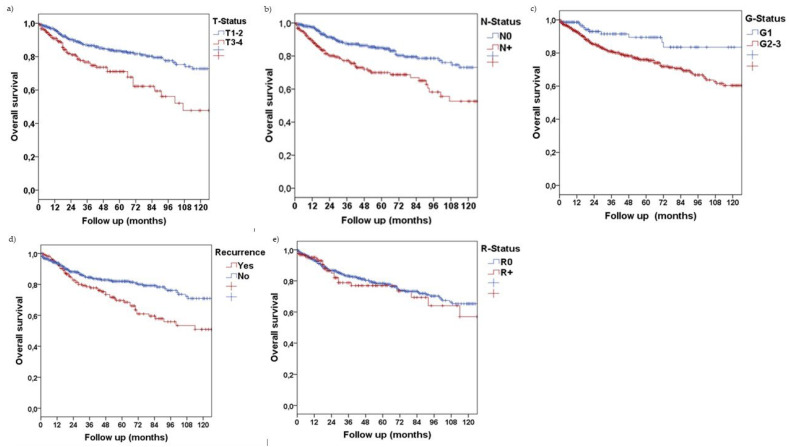
10-year overall survival Kaplan–Meier curves for (**a**) tumor size, (**b**) lymph node status, (**c**) histopathological grading, (**d**) recurrence, (**e**) resection margins.

For overall survival of tumor patients, Kaplan–Meier plots and log rank Mantel–Cox regression displayed that T-status (T1/2 vs. T3/4, *p* < 0.001), N-status (N0 vs. N+, *p* < 0.001), grading (G1 vs. G2/3, *p* = 0.001) as well as tumor recurrence (*p* = 0.002) had a statistically significant influence. Resection status (*p* = 0.310) did not correlate with patients’ outcomes (Figure 2a–e).

## 4. Discussion

Within this large retrospective study, recurrence pattern of patients with oral squamous cell carcinoma were analyzed. It was demonstrated that recurrence is frequent, especially in the first six months after primary tumor diagnosis indicating a need for close follow-up intervals. Furthermore, the key recurrence detection method is clinical examination and histopathological grading may be feasible for risk stratification of tumor recurrence.

Tumor recurrence significantly impacts morbidity and mortality of patients with OSCC; by approximately 64%, with local recurrences being frequent and representing the main cause of death associated with a higher risk of mortality than in other head and neck sites [13]. Therefore, the primary goal of follow-up is a clinical and radiological examination (routinely via CT scan) of the oral cavity and neck to exclude newly developing cancers [12]. However, no international consensus exists for a post-therapeutic surveillance schedule for OSCCs [14].

In a study by Loeffelbein et al., a detailed recommendation for follow-up interval was proposed of every six weeks during the first half-year by single clinical and alternating clinical/radiological check-ups (in combination with computed tomography (CT) or magnetic resonance imaging (MRI)), followed by extended interval with clinical and radiological check-ups of three months in the second half-year. In year two, a similar regimen with a follow-up interval of three months with single clinical and alternating clinical/radiological check-ups is proposed. In year three, a clinical/radiological screening every six months is suggested as being adequate. From year five onwards, an interval of 6–12 months for clinical and radiological check-ups, depending on patient risk factors and disease progression, is recommended [14]. Nonetheless, this regime references the approach of a single center institution and valid data are missing in the manuscript to underpin this sequence proposal. An audit in the United Kingdom revealed that all questioned units examined patients monthly for the first year, and 90% of patients were seen two-monthly for the following year. However, no information about the detected recurrence rate was given. The authors conclude that implementation of a risk-adapted follow-up protocol is generally low [15].

In this study, recurrence rate of 28% was comparable to the literature [2]. Mean time interval from initial treatment to recurrence was 24 ± 26 months with presentation of tumor recurrence in 24% of all recurrences after 12 months. This is in accordance with the literature where a range of time interval of recurrence between 1 month at the earliest and 61 months at the latest is described [3,16]. Within a review by Liu et al., three studies [3,17,18] showed high occurrence rates within the first three months postoperatively [19]. Therefore, a close follow up with monthly presentation of the patient could be deduced in addition to existing guideline recommendations [8]. Figure 1 shows the time points when recurrences were diagnosed. The grey bars show the recommended time points for follow up examination, according to the German guideline for treatment of OSCC [8]. This indicates that with a follow-up examination only every three months, 59% (64 of 109) of the recurrence patients were detected later than with a monthly follow up. This could have led to serious progress of the recurrence and might be associated with a worse prognosis and strengthens the claim for a close follow-up. Nevertheless, there are some arguments that close follow-up calls may be hard to implement in the clinical routine. A major point of criticism is the organizational barriers that may occur in a primary care center. An aggressive follow-up schedule requires personnel other than the senior operating surgeon. Furthermore, patients may find regular reviews tiring and they may not attend due to a multitude of reasons, inter alia, financial limitations [19,20]. If they attend, urgent consultations result in long waits for routine visits and may strengthen discouragement and inhibit adherence. However, the presented data underline the importance of frequent clinical examination as the primary detection method for recurrences. In contrast, Zätterström et al. suggest that no close structured follow-up is needed as self-reported symptoms lead to diagnosis in the majority of tumor recurrences with no difference in disease-free survival in comparison to physical examination of asymptomatic patients. This way, follow-up may promise to be more cost-efficient with trained personnel in collaboration with head and neck specialists that handle parts of follow-up routines [6]. However, the authors did not evaluate imaging methods implemented in the routine follow-up, such as CT or ultrasonography, to detect early recurrence in asymptomatic patients. Especially in the light of the fact that the authors show significantly reduced salvage rate for treatment of recurrence after the first year of follow-up [6], check-up intervals should not solely rely on symptom reports. In the presented analysis, only 2% of recurrence detection was due to self-reported symptoms in this patient cohort. Thus far, additional chair-side tests to improve clinical examination especially in detection of primary but as well as recurrences of oral cancer are subject to several studies. Here, toluidine blue test and fluorescence visualization may be useful to clinically detect occult lesions in the progression pathway to oral cancer, but is not routinely performed [21,22,23]. Discussion of which intervals and specific diagnostic methods should be applied for radiological follow-up of tumor patients is an ongoing debate [14,24] and was not the primary subject of this investigation. As for radiographic analysis, 27% of the recurrences were primarily detected with CT with contrast of the head and neck. In this study, no PET-CT was performed to evaluate distant metastases (*n* = 4 metastases were detected with CT scan with contrast of the thoracic and abdominal region). In general, PET-CT can be indicated in cases of tumor surveillance if the clinical/radiological suspicion of distant metastases cannot be ruled out nor confirmed via CT scan [25]. Furthermore, PET-CT can offer an additional diagnostic value for the evaluation of the symptomatic patient suspected of having local recurrence, but is not the standard radiological analysis [25,26].

For another approach to facilitate follow-up, a specific risk stratification of patients that bear higher hazard of tumor recurrence may be useful. Here, multiple approaches are demonstrated in the literature [27,28,29]. In this study, the histopathological grading was found to be associated with early tumor recurrence. Consistent with the presented results, Safi et al., confirmed histological grading as an independent risk factor for regional recurrence of OSCC in multivariate analysis within a large retrospective analysis, consisting of 517 patients. The authors concluded that grading needs to be considered for individualized therapy management [30]. In accordance, Xu et al., identified pathological grade as an independent risk factor for early-stage oral squamous cell carcinoma, but not for advanced stage [31]. In addition, other studies stated that the degree of differentiation among other risk factors, such as tumor size and lymph node status, were significantly associated with early tumor recurrence [32,33]. Troeltzsch et al., found histological grading predictive for cervical lymph node metastasis in tumors of the maxillary mucosa [34]. In summary, the histopathological grading may bear the potential for a risk-stratified follow-up regimen that should be considered in further prospective trials.

In addition, follow-up is essential to detect secondary cancers. Patients with cancer of the oral cavity and pharynx have been described to be particularly susceptible to the development of secondary cancers [35]. In general, the annual incidence of secondary cancer after treatment of low stage oral cancer is approximately 3% [36]. There is increasing evidence in the literature that risk stratification with histopathological features analogous to the discussed approach may be useful in early detection of secondary tumors. Occurrences were lower among those with potentially human papillomavirus (HPV)-associated head and neck squamous cell carcinoma [37]. Interestingly, indexed hypopharynx and oropharynx carcinomas showed slightly higher risk for second primary cancers [38]. Given the hypothesis that OSCCs are not predominately associated with HPV [39], future studies may focus on this specific subsite to further evaluate the potential prognostic factor for occurrence of secondary cancer. Despite the epidemiological data of frequent second primary tumors in OSCC patients, questions are raised whether the oral cancer surgeon is the appropriate physician for such diagnostic investigations or if inclusion of regular visits to a primary care physician may be more suitable to cover this aspect of aftercare [19].

This study suffers from some major limitations: first and overall, as a retrospective study, data acquisition depends highly on the accuracy of the clinical records. In addition, patients may become lost to follow-up. Next, although potential risk factors for OSCC, such as nicotine or alcohol abuse, were documented within the scope of initial staging, no interrogation of patients’ smoking or drinking behaviors was done after treatment that might influence tumor recurrence as a confounder. Finally, margin dysplasia, considered by the WHO as an influencing factor for local recurrence, was not evaluated in this study. Therefore, close margin resection may act as a confounder and could explain why resection status did not influence patients’ outcomes.

## 5. Conclusions

In summary, tumor recurrence was frequent and manifestation within the first 6–12 months was high, indicating close follow-up regimens, at least during the first year. Clinical examination was the method of choice to detect tumor recurrence, self-reported symptoms led to a minority of recurrence diagnoses. To optimize check-up intervals, risk stratification may be useful and the degree of histopathological grading was shown as an often-underestimated possible prognostic factor for tumor recurrence. Future prospective, longitudinal studies should be initiated to obtain evidence of high quality that may help to translate this hypothesis into clinical considerations.

## Figures and Tables

**Table 1:** Epidemiological data of the patients analyzed in this study (*n* = 760).

		*n* = 760
Patients	Mean age	62 ± 13 years
	Female	*n* = 258 (34%)
	Male	*n* = 502 (66%)
Risk factors	No risk factors Smoking and alcohol abuse Smoking Alcohol abuse	*n* = 191 (25%) *n* = 374 (49%) *n* = 96 (13%) *n* = 99 (13%)
Primary tumor sites	Floor of the mouth Tongue Mandible Maxilla Cheek	*n* = 226 (30%) *n* = 209 (27%) *n* = 167 (22%) *n* = 111 (15%) *n* = 47 (6%)
Primary Tumor stadium (UICC, = Union for International Cancer Control)	Tumor stadium 1 Tumor stadium 2 Tumor stadium 3 Tumor stadium 4	*n* = 263 (33%) *n* = 144 (17%) *n* = 91 (12%) *n* = 319 (38%)
Primary Tumor size	T1 T2 T3 T4	*n* = 311 (41%) *n* = 231 (30%) *n* = 43 (6%) *n* = 175 (23%)
Primary lymph node status	N0 N1 N2a N2b N2c	*n* = 502 (66%) *n* = 98 (13%) *n* = 6 (1%) *n* = 111 (15%) *n* = 43 (5%)
Histopathological grading	G1 G2 G3	*n* = 125 (17%) *n* = 541 (71%) *n* = 94 (12%)
Resection margin	R0 R1 R2	*n* = 643 (85%) *n* = 110 (14%) *n* = 7 (1%)
Therapy of primary tumor	Surgery only Adjuvant radiation Adjuvant radiochemotherapy	*n* = 423 (56%) *n* = 146 (19%) *n* = 191 (25%)

**Table 2:** Epidemiological data of recurrence cases (*n* = 216).

Tumor recurrence	Mean recurrence interval No recurrence Local recurrence Lymph node metastases Distant metastases Second oral cavity cancer combination	*n* = 216 (28%) 24 ± 26 months *n* = 544 (72%) *n* = 75 (10%) *n* = 50 (7%) *n* = 26 (3%) *n* = 58 (7%) *n* = 7 (1%)
Recurrence number in each patient	*n* = 0 *n* = 1 *n* = 2 *n* = 3 *n* = 4	*n* = 544 (72%) *n* = 181 (24%) *n* = 32 (4%) *n* = 2 (<1%) *n* = 1 (<1%)
Tumor recurrence therapy	Surgery only Either radio-, chemo- or both therapies Best supportive care	*n* = 127 (59%) *n* = 58 (27%) *n* = 31 (14%)
Detection of tumor recurrence	Clinical examination Radiological diagnosis Ultrasonography Self-reported symptoms	*n* = 123 (57%) *n* = 57 (27%) *n* = 18 (8%) *n* = 18 (8%)

## Data Availability

Not applicable.
